# A Histological Study of *Aspergillus flavus* Colonization of Wound Inoculated Maize Kernels of Resistant and Susceptible Maize Hybrids in the Field

**DOI:** 10.3389/fmicb.2018.00799

**Published:** 2018-04-24

**Authors:** Gary L. Windham, William P. Williams, J. E. Mylroie, Cedric X. Reid, Erika D. Womack

**Affiliations:** ^1^Corn Host Plant Resistance Research Unit, United States Department of Agriculture-Agricultural Research Service, Mississippi State, MS, United States; ^2^Department of Biochemistry, Molecular Biology, Entomology, and Plant Pathology, Mississippi State University, Mississippi State, MS, United States

**Keywords:** *Aspergillus flavus*, corn (*Zea mays*), maize, aflatoxins, histology, pathogenesis, resistance

## Abstract

*Aspergillus flavus* colonization in developing kernels of maize single-cross hybrids resistant (Mp313E × Mp717) and susceptible (GA209 × T173) to aflatoxin accumulation was determined in the field over three growing seasons (2012–2014). Plants were hand pollinated, and individual kernels were inoculated with a needle dipped in a suspension of *A. flavus* conidia 21 days after pollination. Kernels were harvested at 1- to 2-day intervals from 1 to 21 days after inoculation (DAI). Kernels were placed in FAA fixative, dehydrated, embedded in paraffin, sectioned, and stained with toluidine blue. Kernels were also collected additional kernels for aflatoxin analyses in 2013 and 2014. At 2 DAI, *A. flavus* hyphae were observed among endosperm cells in the susceptible hybrid, but colonization of the endosperm in the resistant hybrid was limited to the wound site of the resistant hybrid. Sections of the scutellum of the susceptible hybrid were colonized by *A. flavus* by 5 DAI. Fungal growth was slower in the resistant hybrid compared to the susceptible hybrid. By 10 DAI, *A. flavus* had colonized a large section of the embryo in the susceptible hybrid; whereas in the resistant hybrid, approximately half of the endosperm had been colonized and very few cells in the embryo were colonized. Fungal colonization in some of the kernels of the resistant hybrid was slowed in the aleurone layer or at the endosperm-scutellum interface. In wounded kernels with intact aleurone layers, the fungus spread around the kernel between the pericarp and aleurone layer with minimal colonization of the endosperm. Aflatoxin B_1_ was first detected in susceptible kernel tissues 8 DAI in 2013 (14 μg/kg) and 2014 (18 μg/kg). The resistant hybrid had significantly lower levels of aflatoxin accumulation compared to the susceptible hybrid at harvests 10, 21, and 28 DAI in 2013, and 20 and 24 DAI in 2014. Our study found differential *A. flavus* colonization of susceptible and resistant kernel tissues, and that the aleurone and the outer layer of the scutellum slowed the rate of colonization by *A. flavus*.

## Introduction

Preharvest infection of maize (*Zea mays* L.) kernels by *Aspergillus flavus* Link:Fr and subsequent accumulation of aflatoxin is a chronic economic and food safety problem in the southeastern United States and is a sporadic problem in the midwestern United States corn belt (Payne, 1992; Park and Liang, 1993; Widstrom, 1996; Wu, 2015). It is estimated that aflatoxins cause an annual loss of $163 million to maize producers in the United States (Wu, 2015). Aflatoxin contaminations of maize is also a problem for maize growers in Asia, sub-Saharan Africa, and in Europe (Darwish et al., 2014; Wu, 2015; Battilani et al., 2016; Agbetiameh et al., 2018). Aflatoxin contamination as a preharvest problem in maize was first reported in 1975 and 1976 (Anderson et al., 1975; Lillehoj et al., 1975). Aflatoxin is one of the most potent carcinogens produced in nature and is commonly found in maize kernels infected with *A. flavus* (Castegnaro and McGregor, 1998). Consumption of aflatoxin-contaminated foods is a major cause of liver toxicity and hepatocellular carcinoma, the fifth most common cancer in the world (Wild and Hall, 2000; Wang et al., 2001; Ayeni, 2015). It has been estimated that five billion people worldwide are exposed to dietary aflatoxin on a regular basis (Wu, 2015). Individuals exposed to both high levels of aflatoxin and chronic hepatitis B virus infection have an increased risk of liver cancer (Liu and Wu, 2010). The U.S. Food and Drug Administration limits the sale of grain with aflatoxin levels exceeding 20 μg/kg. Grain exceeding 20 μg/kg cannot be shipped across state lines and can only be used for livestock feed. Aflatoxin contaminated grain not exceeding 100, 200, or 300 μg/kg can be used as feed for breeding beef cattle and swine, finishing swine, or finishing beef cattle in the United States, respectively. Globally, over 100 nations worldwide have established maximum tolerable levels for aflatoxin in food ranging from 2 to 10 μg/kg (Wu, 2015).

Plant resistance is considered to be one of the most desirable strategies for managing aflatoxin accumulation in maize. The development of aflatoxin resistant germplasm has been the primary emphasis of a number of research organizations, particularly the United States Department of Agriculture-Agricultural Research Service (USDA-ARS) (Scott and Zummo, 1990, 1992; Williams and Windham, 2001, 2006; Williams et al., 2015). The USDA-ARS Corn Host Plant Resistance Research Unit located at Mississippi State, MS, United States, released the first two maize lines (Mp313E, Mp420) with resistance to kernel infection by *A. flavus* (Scott and Zummo, 1990, 1992). More recently, four additional germplasm lines (Mp715, Mp717, Mp718, Mp719) with resistance to aflatoxin accumulation have been released (Williams and Windham, 2001, 2006, 2012). Progress has been made in identifying molecular markers associated with resistance to aflatoxin accumulation in our lines (Brooks et al., 2005; Warburton et al., 2009, 2011). Studies have also been conducted to determine cell constituents and metabolic pathways associated with *A. flavus* resistance in maize (Hawkins et al., 2015; Tang et al., 2015). However, little information is available on physical barriers inside the kernels that contribute to host resistance mechanisms in these germplasm.

The objective of our study were to compare colonization of *A. flavus* in maize resistant and susceptible to aflatoxin accumulation in order to identify sites within developing kernels that are associated with limiting fungal spread.

## Materials and Methods

### Fungal Strain and Inoculum Production

*Aspergillus flavus* strain NRRL 3357, which is known to produce aflatoxin in maize grain (Windham and Williams, 1998, 2002), was used as inoculum. Cultures were started each spring using freeze-dried plugs supplied by the USDA-ARS Bacterial Food-borne Pathogen and Mycology Research Unit (Peoria, IL, United States). Inoculum was increased on sterile corn cob grits (Grit-O-Cobs^®^, The Andersons Inc., Maumee, OH, United States) in 500-ml flasks, each containing 50 g of grits and 100 ml of sterile, distilled water, and incubated at 28°C for 3 weeks. Conidia in each flask were washed from the grits using 500 ml sterile distilled water containing 20 drops of Tween 20 (Sigma-Aldrich, St. Louis, MO, United States, Cat. # P1379) per liter and filtered through four layers of sterile cheesecloth. Inoculum not used immediately was refrigerated at 4°C. Fresh *A. flavus* inoculum was collected each week.

### Maize Kernel Production, Inoculation, and Harvest

**FIGURE 1:**
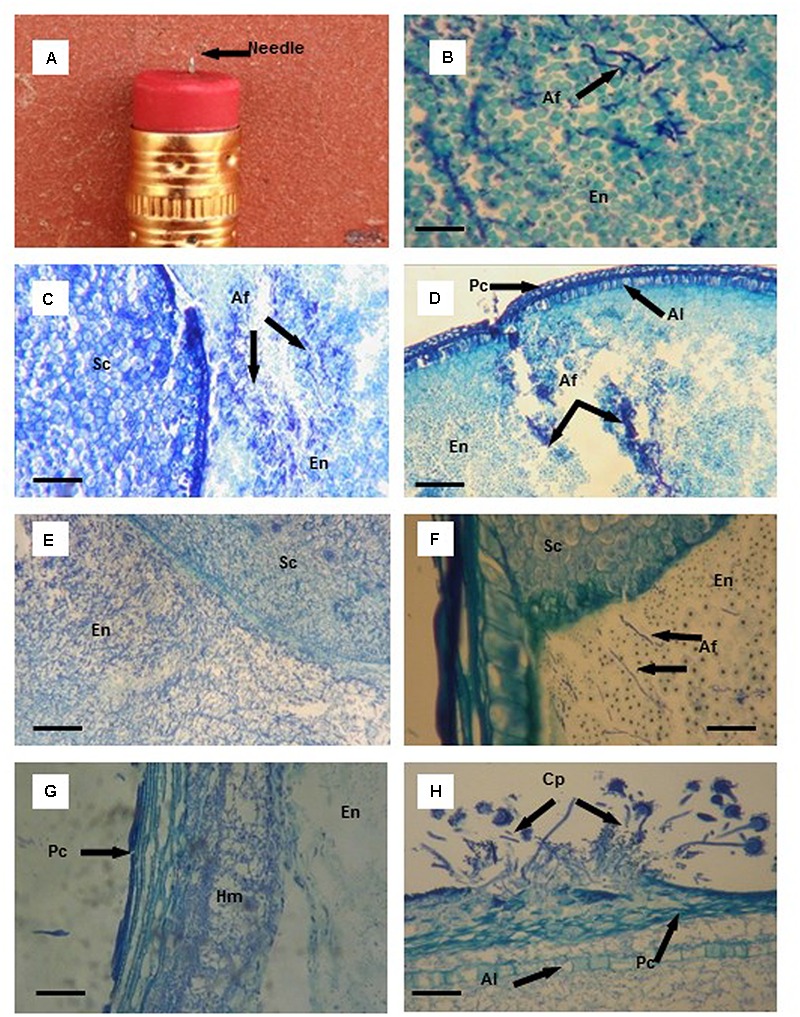
Thin tissue sections **(B–H)** from aflatoxin resistant (Mp313E × Mp717) and susceptible (Ga209 × T173) maize kernels infected with *Aspergillus flavus* (Af) using a needle **(A)** imbedded in a pencil eraser as the artificial inoculation device. **(B)** Initial colonization of endosperm (En) tissues by *A. flavus* hyphae (black arrows). **(C)** Colonization of endosperm (En) tissue of the susceptible hybrid by *A. flavus* 3 days after inoculation (DAI); no fungal colonization of the scutellum (Sc). **(D)** *A. flavus* hyphae concentrated at the wound of the endosperm (En), aleurone layer (Al), and pericarp (Pc) in the resistance hybrid 3 DAI. **(E)** Cells containing starch granules disappear with extensive colonization of the endosperm (En) and scutellum (Sc) of the susceptible hybrid by *A. flavus* 5 DAI. **(F)** *A. flavus* hyphae advance toward the scutellum (Sc) through the endosperm (En) in the resistant hybrid. **(G)** *A. flavus* hyphal mat (Hm) formed between the pericarp (Pc) and the outer layer of the endosperm (En) 6 DAI. **(H)** *A. flavus* conidiophores (Cp) found on the kernel surface after the fungus had erupted through the pericarp (Pc) 7 DAI; extensive *A. flavus* colonization between the pericarp and aleurone layer (Al), and in the endosperm. Scale bars: **(B)** 30 μm; **(F)** 50 μm; **(G,H)** 100 μm; **(C–E)** 200 μm.

An *A. flavus* resistant single-cross maize hybrid (Mp313E × Mp717) and a susceptible single-cross maize hybrid (GA209 × T173) were grown at the R. R. Foil Plant Science Research Center located at Mississippi State University, MS, United States, in 2012–2014. The inbred parents (Mp313E and Mp717) of the resistant hybrid typically have lower levels of aflatoxin accumulation and *A. flavus* colonization when compared with inbred parents (GA209 and T173) of the susceptible hybrid in field trials (Scott and Zummo, 1990; Williams and Windham, 2006; Brown et al., 2016). Plants were hand pollinated and covered with a paper pollination bag (Lawson 402, Northfield, IL, United States). Individual kernels were inoculated 21 days after pollination with a size 12 quilting needle (Entaco Limited, Worcestershire, England, Cat.# JJ12012) imbedded in a pencil eraser with 1 mm of the pointed tip exposed (**Figure 1A**). A scalpel was used to make a 2.5 cm cut across the ear husks, and then two longitudinal cuts 5.0 cm in length were made. The husks were peeled back to expose the developing kernels. Individual kernels were inoculated by dipping the needle tip in a suspension of *A. flavus* conidia (1 × 10^8^ conidia/ml) and inserting the needle into the center of the exposed crown of the kernel. Husks were repositioned over the kernels, secured with a rubber band, and the ear was covered with a clear plastic bag. To examine the potential for *A. flavus* movement into adjacent, uninoculated kernels, eight kernels surrounding a selected kernel were inoculated with *A. flavus* as described above. Developing kernels are attached to the cob in paired rows and share vascular tissues that connect to the main vascular bundle in the cob (Smart et al., 1990). By inoculating kernels in rows adjacent to the uninoculated kernels, it would allow us to determine if it was possible for *A. flavus* to colonize adjacent, uninoculated kernels via the vascular tissue of the cob. For histological studies, three ears of each hybrid were harvested at 1- to 2-day intervals from 1 to 21 days after inoculation (DAI). Four to five inoculated kernels from each ear were placed in vials containing the fixative FAA (formalin-acetic acid-alcohol) (Berlyn and Miksche, 1976) and kept in a refrigerator at 4°C.

### Histological Methods

Kernels were removed from the FAA fixative, dehydrated through a graded tertiary butyl alcohol series, and embedded in Para last Plus wax (Sigma-Aldrich, St. Louis, MO, United States, Cat. #P3683) (Berlyn and Miksche, 1976). Tissue sections (10 μm) were cut longitudinally from the wax-embedded kernels using an American Optical Rotary microtome, affixed to glass microscope slides with Haupt’s gelatin adhesive, and stained with 0.05% aqueous toluidine blue. Cover slips were cemented over stained sections using a Permount mounting media (Fisher Scientific, Fair Lawn, NJ, United States, Cat. #SP15-100), and viewed with an Olympus BH-2 compound microscope.

### Aflatoxin Quantification

In 2013 and 2014, additional kernels for aflatoxin analyses (four to five from each ear) were collected from the ears harvested for the histological study. Aflatoxin B_1_ is the most common aflatoxin produced by aflatoxin producing *Aspergillus* species (Mehl and Cotty, 2010) and was the only aflatoxin quantified. In 2013, wound-inoculated and uninoculated kernel samples were collected 2, 4, 6, 8, 9, 10, 12, 14, 21, and 28 DAI and prepared for aflatoxin analyses using methodology described by Reid et al. (2016). Briefly, kernels were flash frozen in liquid nitrogen and ground into a fine powder with a mortar and pestle. Ground samples (200 mg) were placed in 2.0 ml micro-centrifuge tubes and 1.0 ml of (70/30, v/v) methanol/water was added to each tube and vortexed for 1 min. Tubes were centrifuged at 14,000 rpm for 5 min. The supernatant was collected and passed through polytetrafluoroethylene syringe filters (0.45 μm). The liquid extracts were transferred to auto-sampler vials and aflatoxin B_1_ was quantified using an Agilent 6460 Triple Quadruple tandem mass spectrometer (Reid et al., 2016). Recovery of aflatoxin B_1_ in spiked samples was 90%.

In 2014, kernel samples collected daily 3–10 DAI, and then every other day from 12 to 24 DAI. Kernels were flash frozen in liquid nitrogen and ground with a 2000 Geno/Grinder (SPEX Certiprep, Metuchen, NJ, United States) for 3 min at 1200 strokes/min. Aflatoxin was extracted from the ground samples and the supernatant treated as described above. Samples were analyzed for aflatoxin B_1_ on an Agilent 1100 Infinity Liquid Chromatography (HPLC) system coupled to fluorescence detector (excitation 362 nm, emission 455 nm). Analysis was performed by injecting 10 μL of the sample onto a Zorbax Eclipse XDB-C18 4.6 mm × 150 mm, 3.5 μm column from Agilent (Santa Clara, CA, United States) with a column temperature of 25°C. The mobile phase consisted of HPLC grade water, methanol, and acetonitrile (50:40:10 v/v/v) and was pumped at a flow rate of 0.8 ml/min under isocratic conditions for a total run time of 10 min. Linearity was evaluated with a matrix-matched 10-point calibration curve (replicated twice) ranging from 3.9 to 2,000 μg/kg aflatoxin B_1_. The least square method was used to estimate the linear regression. A satisfactory linear correlation was determined by the coefficient of determination (*R*^2^). Accuracy of the extraction method was evaluated by performing recovery experiments using ground samples of uninoculated corn kernels spiked with 50 μg/kg aflatoxin B_1_ (*n* = 3). Recovery of aflatoxin B_1_ in spiked samples was 93%.

### Statistical Analyses of Aflatoxin Data

All statistical analyses were conducted using the SAS software package (version 9.4; SAS Institute Inc., Cary, NC, United States). Means for aflatoxin concentration were transformed [ln(*y*+1)] before statistical analyses. Transformed data was analyzed using the Proc GLM procedure to compare aflatoxin contamination of the resistant and susceptible hybrids for individual harvest dates.

## Results

One advantage our study has over similar studies is that the USDA-ARS maize breeding program has developed aflatoxin resistant maize genotypes through conventional breeding methods. These resistant genotypes can be compared with susceptible genotypes to determine the physical, chemical, and genetic factors that affect *A. flavus* growth and subsequent aflatoxin production in developing kernels. In our present study, we found differences in *A. flavus* growth and aflatoxin accumulation in the inoculated kernels of the resistant and susceptible maize hybrids.

### Colonization of Kernels by *A. flavus*

**FIGURE 2:**
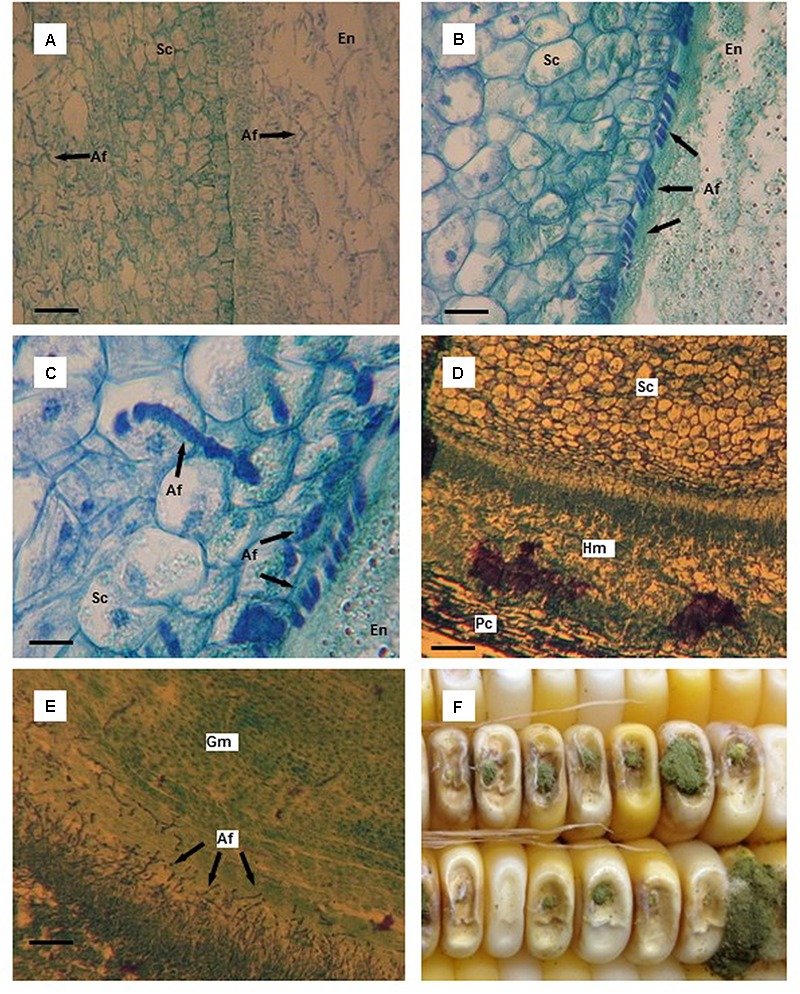
Thin tissue sections **(A–E)** and whole kernels **(F)** of aflatoxin susceptible and resistant maize hybrids colonized by *Aspergillus flavus*. **(A)** *A. flavus* colonized endosperm (En) and scutellum (Sc) of the susceptible hybrid 10 days after infection (DAI); starch granules in the endosperm have disappeared and are replaced by mycelium. **(B)** *A. flavus* hyphae massed at the endosperm (En)-scutellum (Sc) interface in the resistant hybrid 10 DAI; notice the outer, organized layer of cells in the scutellum. **(C)** *A. flavus* penetration of scutellum (Sc) cells in the resistant hybrid 10 DAI. **(D)** *A. flavus* hyphal mat found between the pericarp (Pc) and the aleurone layer adjacent to the scutellum (Sc) 12 DAI in the susceptible hybrid. **(E)** *A. flavus* invasion of germ (Gm) tissue of the susceptible hybrid 12 DAI. **(F)** Typical *A. flavus* growth and sporulation on needle-inoculated kernels. Scale bars: **(C)** 25 μm; **(B)** 50 μm; **(D)** 75 μm; **(A,E)** 100 μm.

Toluidine blue stained *A. flavus* hyphae (**Figure 1B**) a dark blue, and provided excellent contrast between the fungal hyphae and kernel tissues in the endosperm, scutellum, and germ. The infection processes described in **Figures 1C–H** and **2A–E** summarize *A. flavus* colonization observed in 2012 and 2013. Unseasonably cooler weather (mean ambient temperatures were 4°C lower) during early stages of kernel infection in 2014 delayed kernel colonization compared to fungal spread observed in 2012 and 2013. In general, *A. flavus* initially colonized the starchy endosperm and the area between the pericarp and aleurone in the wound inoculated kernels. Thus, the fungus began a two-pronged approach attacking the scutellum first and finally the germ.

No *A. flavus* hyphae were observed in kernel tissues 24 h after inoculation in either of the maize hybrids. By two DAI, small clumps of hyphae were found in the endosperm of the susceptible hybrid and within the wounded area in the endosperm of the resistant hybrid. *A. flavus* had colonized areas of the endosperm away from the infection site in the susceptible hybrid 3 DAI (**Figure 1C**); however, hyphae remained concentrated near the wound damaged area in the endosperm of the resistant hybrid (**Figure 1D**). In the susceptible hybrid 5 DAI, there was extensive fungal colonization of the endosperm and the scutellum (**Figure 1E**). Starch granules in the endosperm began to disappear as the amount of mycelial growth increased. In the resistant hybrid 5 DAI, *A. flavus* had colonized a smaller amount of the endosperm compared to the area colonized in the susceptible hybrid, and had not invaded the scutellum (**Figure 1F**). Endosperm cells containing starch granules were still intact.

Our needle inoculator wounded the kernel through the pericarp and the aleurone; the fungus was provided with an entrance to invade the space between these two outer layers of the seed. As the fungus spread between these two layers, a hyphal mass formed separating the pericarp and the aleurone layer (**Figure 1G**). This invasion route provided the fungus with access to other areas of the developing seed. It was not uncommon for the aleurone layer to remain intact as *A. flavus* spread between the two layers. In one wound-inoculated kernel of the susceptible hybrid, the pericarp was penetrated by the needle inoculator, but the aleurone layer was not broken. *A. flavus* spread 95% of the way around the seed by 6 DAI solely by moving between the pericarp and aleurone layer. As *A. flavus* hyphae spread around the kernel, only a very small area of the endosperm and scutellum were invaded by the fungus.

Sporulation of *A. flavus* was visible on the outer surface of the wound-inoculated kernels beginning 4–5 DAI. Typically, the first conidiophores observed were produced by mycelium growing at the wound site. But additional areas of conidiophore production were observed externally on the kernel surface when the fungus erupted through the pericarp (**Figure 1H**).

By 10 DAI in the kernel tissue of the susceptible hybrid, mycelium had ramified the endosperm and very few starch granules were visible (**Figure 2A**). Essentially, the starchy endosperm had been replaced by a mass of *A. flavus* mycelium. Also, the scutellum was heavily colonized by the fungus. In a resistant kernel 10 DAI, *A. flavus* hyphae had spread through the endosperm and were found concentrated at the endosperm-scutellum interface in a somewhat orderly manner (**Figure 2B**). The outer layer of the scutellum had limited the amount of infection. Very few areas of the scutellum were infected by the fungus which had only grown 2–4 layers deep into the scutellum. The penetrating hyphae appeared to be constricted as the fungus penetrated cell walls in the scutellum (**Figure 2C**).

The scutellum in the susceptible hybrid kernels was heavily colonized by 12 DAI via the scutellum-endosperm interface and also by fungal spread around the kernel between the pericarp and aleurone layer. The scutellum was invaded from a hyphal mass that formed between the pericarp and aleurone layer adjacent to the scutellum and opposite the endosperm (**Figure 2D**). The germ was also infected by *A. flavus* from the endosperm (**Figure 2E**). At later harvest dates, the scutellum and the germ in the kernels of the resistant hybrid were invaded by *A. flavus*. Conidiophore development externally increased through the last harvest dates, but was somewhat less in the resistant hybrid compared to the susceptible hybrid (**Figure 2F**).

In 2012 and 2013, uninoculated kernels that were paired with *A. flavus* inoculated kernels (all surrounding kernels had been inoculated to insure the infection of one of the paired kernels) were harvested at 7, 14, and 21 DAI to determine fungal spread via the rachilla (shared vascular tissue). *A. flavus* was not found internally in any of the uninoculated kernels even though these kernels were surrounded by kernels covered with abundant *A. flavus* mycelia. Fungal growth was also not observed in controlled, uninoculated kernels harvested at various dates during the study.

### Aflatoxin Accumulation

In 2013, aflatoxin was first detected (14 μg/kg) in kernels of the susceptible hybrid 8 DAI (**Table 1**). Aflatoxin levels in the susceptible hybrid increased each harvest date, with the exception of 12 DAI, until the last harvest date (28 DAI). Aflatoxin was only detected in the kernels of the resistant hybrid at one harvest date (14 DAI). Aflatoxin accumulation in the infected kernels at 8 and 10 DAI would have coincided with extensive infection of the endosperm and, in later harvest dates (21 and 28 DAI) aflatoxin accumulation would have coincided with infection of the scutellum and germ. Significantly higher levels of aflatoxin contamination were found in the susceptible hybrid than in the resistant hybrid 10, 21, and 28 DAI.

**Table 1:** Aflatoxin accumulation in the developing maize kernels of resistant (Mp313E × Mp717) and a susceptible (GA209 × T173) hybrids inoculated with *Aspergillus flavus* in 2013 (21 days after pollination).

Days after	Aflatoxin (μg/kg)	
Inoculation	GA209 × T173	Mp313E × Mp717
2	0 ± 0	0 ± 0
4	0 ± 0	0 ± 0
6	0 ± 0	0 ± 0
8	14 ± 24	0 ± 0
10	83 ± 71*	0 ± 0
12	0 ± 0	0 ± 0
14	126 ± 159	125 ± 216
21	3376 ± 2631*	0 ± 0
28	1370 ± 583*	0 ± 0

*^∗^An asterisk indicates significant (P ≤ 0.05) differences between susceptible and resistant maize hybrids for individual harvest dates.*

In 2014, aflatoxin was detected in small amounts (1–8 μg/kg) in kernels of the resistant hybrid from 4 to 9 DAI (**Table 2**). Aflatoxin was first found in kernels of the susceptible hybrid 8 DAI. Aflatoxin accumulation at levels lower than 20 μg/kg were detected in kernels of the susceptible hybrid 8, 9, and 10 DAI. Much higher levels of aflatoxin accumulation were found from 12 DAI through the last harvest 24 DAI in kernels of both hybrids. Significantly higher levels of aflatoxin contamination were found in the susceptible hybrid than in the resistant hybrid 20 and 24 DAI. Aflatoxin contamination was highest when the fungus would have invaded most of the endosperm and the scutellum as observed in the kernels processed in the histological part of this study.

**Table 2:** Aflatoxin accumulation in the developing maize kernels of resistant (Mp313E × Mp717) and a susceptible (GA209 × T173) hybrids inoculated with *Aspergillus flavus* in 2014 (21 days after pollination).

Days after	Aflatoxin (μg/kg)	
Inoculation	GA209 × T173	Mp313E × Mp717
3	0 ± 0	0 ± 0
4	0 ± 0	1 ± 2
5	0 ± 0	2 ± 3
6	0 ± 0	2 ± 4
7	0 ± 0	2 ± 3
8	18 ± 9	8 ± 13
9	4 ± 4	2 ± 3
10	10 ± 17	66 ± 45
12	235 ± 261*	643 ± 706
14	2545 ± 738	0 ± 0
16	248 ± 394	150 ± 259
18	1856 ± 1418	578 ± 382
20	4688 ± 748*	146 ± 218
22	1996 ± 1212	254 ± 148
24	5745 ± 1424*	507 ± 717

*^∗^An asterisk indicates significant (P ≤ 0.05) differences between susceptible and resistant maize hybrids for individual harvest dates.*

## Discussion

Working with a weak pathogen such as *A. flavus* in pathological studies on developing maize kernels in the field is a challenge. Although a number of researchers have reported probable routes of *A. flavus* infection in developing kernels (Marsh and Payne, 1984a,b; Smart et al., 1990; Payne, 1992), it can be difficult to achieve adequate levels of *A. flavus* infection of kernels in field studies even when placing high numbers of conidia on external silks or inside ear husks (Windham et al., 2009). Thus, it was necessary for us to artificially inoculate developing kernels with *A. flavus* with our needle inoculator. In the field, maize ears are subjected to various environmental stresses (Odvody et al., 1997) and insect feeding (McMillian et al., 1980, 1985, 1990; Steffey et al., 1999; Windham et al., 1999) which damage the pericarp and provide *A. flavus* entry into the kernel. The needle inoculation method used in this study, in most cases, penetrated the seed pericarp and aleurone layer in the exposed crown end of the kernel similar to damage caused by insect probing and feeding.

In our study, the starting point of *A. flavus* infection of the inoculated kernels was in the wound through the pericarp, aleurone layer, into the endosperm. Researchers have found that *A. flavus* colonizes the endosperm to different degrees (Smart et al., 1990; Keller et al., 1994; Brown et al., 1995). In our study, the fungus readily colonized the starchy endosperm of the inoculated kernels. Our observations were similar to those of Dolezal et al. (2013) and Shu et al. (2015) although *A. flavus* colonized the endosperm at a slower rate in kernels of the resistant hybrid compared to the susceptible hybrid. Endosperm-specific antifungal proteins (PR-10, a 14-kDa trypsin inhibitor, and resistance-associate proteins) have been found in aflatoxin resistant germplasm and may explain the slower growth of *A. flavus* in kernels of the resistant hybrid (Chen et al., 2015).

Another route of kernel colonization by *A. flavus* is between the pericarp and aleurone layers. In our study, the fungus grew between these layers and formed a thickened hyphal mat within 5–7 DAI. Dolezal et al. (2013) observed minimal growth of *A. flavus* between the pericarp and aleurone layer toward the scutellum or germ 4 DAI. However, we found that *A. flavus* colonization between these layers was extensive and by 10–12 DAI the fungus was beginning to colonize the germ and scutellum on the side opposite the endosperm via this route. Our findings agree with those of Brown et al. (1995) in which they found that the scutellum region could easily be colonized by *A. flavus* from the aleurone. One of the significant findings we observed was the extensive movement of *A. flavus* between the pericarp and aleurone. In a kernel of the susceptible hybrid collected 6 DAI, our inoculation with the needle did not penetrate the aleurone layer. The fungus had almost completely encircled the kernel between the pericarp and aleurone 6 DAI. The aleurone was still intact, and there was very minimal colonization of the endosperm or the scutellum. The intact aleurone served as a barrier limiting *A. flavus* colonization of the starchy endosperm and scutellum. In cereals, the aleurone layer is the outer layer of the endosperm and contains protein bodies and enzymes associated with endosperm digestion (Esau, 1977). The aleurone has also been found to contain antifungal proteins including a ribosome-inactivating protein (Guo et al., 1999).

Differences in colonization of the scutellum in kernels of the resistant and the susceptible hybrids were observed in our studies. In the susceptible hybrid, the scutellum was colonized at the endosperm-scutellum interface 4–5 DAI. In some cases, the *A. flavus* hyphae would mass outside the scutellum. Dolezal et al. (2013) and Shu et al. (2015) described the fungal mass as a biofilm which consisted of highly branched, tightly intertwined hyphae which pressed against the scutellum. This biofilm was not always necessary for infection of the scutellum, but was very common at the endosperm-scutellum interface. In our study, *A. flavus* also formed a similar structure at the endosperm-scutellum interface. Another significant event we observed, was the formation of an orderly mass of *A. flavus* hyphae at the endosperm-scutellum interface of resistant kernels 10 DAI. The fungal hyphae lined up at the surface of the scutellum of the resistant hybrid, but not in an extensive mass of hyphae similar to what we observed at the endosperm-scutellum interface in kernels of the susceptible hybrid. By this time point, *A. flavus* colonization of the scutellum in the susceptible hybrid was extensive. However, colonization of the scutellum in the resistant hybrid was limited to 2–4 cells deep. An organized layer of cells surrounding the scutellum appeared to be a barrier to fungal infection. One explanation of this defense mechanism could be that the glandular layer of the scutellum contains antifungal ribosome-inactivation proteins (Guo et al., 1999).

One route of kernel colonization by *A. flavus* we attempted to study was the infection of uninoculated kernels via the pedicel tip where the kernel is attached to the cob. Kernels are paired in their attachment to the cob in that the rachilla of the kernels is attached to the same vascular bundle (Smart et al., 1990). We inoculated all of the kernels surrounding select kernels to insure that the uninoculated kernel’s paired kernel was infected with *A. flavus.* However, *A. flavus* was not found in any of the uninoculated kernels harvested 7, 14, and 21 DAI. Even with copious amounts of fungal hyphae and conidiophores located on adjacent kernels, none of these uninoculated kernels were infected with *A. flavus*. Smart et al. (1990) suggested that a point of *A. flavus* entry into developing kernel was the site of attachment to the cob. However, we did not observe any *A. flavus* colonization in the pedicel region of our paired, uninoculated kernels.

In maize breeding programs developing resistance to *A. flavus*, the accumulation of aflatoxin in developing ears in the field that have been artificially inoculated with this fungus is used to select resistant lines. In field evaluations, the level of *A. flavus* infection is lower in resistant lines compared to susceptible for lines. Since aflatoxin quantification is quicker and more efficient than determining fungal infection, levels of aflatoxin contamination are used by plant breeders and pathologists to identify resistant genotypes. Typically, inoculation techniques are used that cause limited kernel wounding. In our current study, we used a severe wounding technique to insure infection of kernels to be collected for histological purposes. This led to higher levels of aflatoxin contamination in the resistant lines than what we normally find in field evaluations. In our field evaluations, it not uncommon to find aflatoxin levels lower than 20 μg/kg in mature kernels of resistant lines (Williams et al., 2015; Brown et al., 2016).

Environmental conditions such as low rainfall and higher than normal ambient temperatures during kernel development can have a significant effect on aflatoxin accumulation (Windham et al., 2009). In our present study, mean ambient temperatures at time of inoculation in 2013 was 26°C and in 2014 was 22°C. Initially, *A. flavus* colonization of kernel tissues was slowed in both hybrids in 2014. Optimum *A. flavus* growth has been reported at 29°C and optimum aflatoxin production by *A. flavus* was at 25°C (Schindler et al., 1967). As temperatures rose to normal levels during the 2014 experiment, high amounts of aflatoxin were found in the kernel tissues at the latter harvest dates. Aflatoxin contamination in maize field trials can fluctuate year to year as a result of temperature and soil moisture stresses on the plant (Widstrom, 1996; Windham and Williams, 1998, 2002).

Significant findings in our study were: (1) slower *A. flavus* internal colonization of kernels of the resistant hybrid compared to the susceptible hybrid, (2) *A. flavus* readily invaded the scutellum/germ tissue via mycelial growth between the pericarp and aleurone, and (3) the aleurone layer and the outer layer of the scutellum were found to be protective barriers limiting *A. flavus* colonization of the endosperm and scutellum/germ tissues.

## Author Contributions

GW conceived the study, made all inoculations, collected all kernel tissues, and oversaw processing of histological slides. WW produced the seed in the field nursery, planted and maintained the plants, and made all of the pollinations of the test plants. JM, CR, and EW developed the techniques to analyze small kernel samples for aflatoxin and conducted all of the aflatoxin quantifications.

## Conflict of Interest Statement

The authors declare that the research was conducted in the absence of any commercial or financial relationships that could be construed as a potential conflict of interest. The reviewer EG-C and handling Editor declared their shared affiliation.
